# Enhancement of heat transfer and entropy generation analysis of nanofluids turbulent convection flow in square section tubes

**DOI:** 10.1186/1556-276X-6-252

**Published:** 2011-03-24

**Authors:** Vincenzo Bianco, Sergio Nardini, Oronzio Manca

**Affiliations:** 1 Dipartimento di Ingegneria Aerospaziale e Meccanica, Seconda Università degli Studi di Napoli, Via Roma 29, Aversa, CE 81031, Italy

## Abstract

In this article, developing turbulent forced convection flow of a water-Al_2_O_3 _nanofluid in a square tube, subjected to constant and uniform wall heat flux, is numerically investigated. The mixture model is employed to simulate the nanofluid flow and the investigation is accomplished for particles size equal to 38 nm.

An entropy generation analysis is also proposed in order to find the optimal working condition for the given geometry under given boundary conditions. A simple analytical procedure is proposed to evaluate the entropy generation and its results are compared with the numerical calculations, showing a very good agreement.

A comparison of the resulting Nusselt numbers with experimental correlations available in literature is accomplished. To minimize entropy generation, the optimal Reynolds number is determined.

## Introduction

High heat transfer performance is vital in many engineering applications, both at the micro and the macro level (i.e. chip cooling and building heating).

The conventional methods to increase the heat transfer rate are those of extending the exchange surface or using a better fluid. The first approach is usually not preferred, because it leads to an increase of thermal system dimensions. Therefore, the second option is more desirable, but it is constrained by the thermophysical properties of conventional heat transfer fluids (i.e. water, ethylene glycol, etc.).

Over the last several decades, scientists and engineers have tried to develop fluids, which provide better performances for a variety of thermal applications.

Applying nanotechnology to heat transfer, the new concept of 'nanofluid', introduced by Choi [1] in 1995, has been proposed to meet the new heat transfer challenges. This new kind of fluid is manufactured by dispersing an amount of solid nanoparticles in traditional heat transfer fluids.

Maxwell [2] was the first to show the possibility of increasing thermal properties, particularly conductivity, of a liquid by including a volume fraction of solid particles. However, the dimensions of the particles were in the order of millimetre or micrometre, hence problems such as mixture stability and a dramatic increase in mixture viscosity were detected.

Several investigations revealed that the dispersion of a small amount of different kinds of nanoparticles (i.e. Al_2_O_3_, CuO, TiO_2_) in water or ethylene glycol exhibit enhanced thermal conductivity, as reviewed in [3-5].

Different concepts have been proposed to explain this enhancement in thermal performance, which results to be higher with respect to that of classical mixtures.

Li and Xuan [6] and Xuan and Roetzel [7] attributed the enhancement of heat transfer to the increased thermal dispersion resulting from the chaotic movements of nanoparticles, which accelerates the exchange of energy. Keblinski et al. [8] proposed different mechanisms that contribute to the increase of nanofluids heat transfer, among which are Brownian motion of the particles and molecular level layering at the liquid/particle interface. Also Wang et al. [9] explained the heat transfer enhancement with the interface layer between liquid and particles. Buongiorno [10] developed a very in-depth analysis of all the possible mechanisms of fluid particles slip during convection of nanofluids, concluding that the abnormal increase of heat transfer coefficient in turbulent regime is due to the variation of thermophysical properties within the boundary layer, because of the effect of the temperature gradient and thermophoresis.

Many authors [11-16] focused their analysis on the measurement of nanofluid thermal conductivity, showing a much larger value with respect to the classical theoretical predictions [17]. In a recent article, Buongiorno et al. [18] conducted an international benchmark exercise on nanofluid thermal conductivity measurements, which concluded that no anomalous enhancement of thermal conductivity was observed.

Other authors concentrated their research on the experimental analysis of nanofluids forced convection in laminar and turbulent regime. The works of Pak and Cho [19] and Xuan and Li [20] represent two outstanding contributions to the experimental study of turbulent convection of nanofluids. They developed two correlations for the calculations of Nusselt number, indicating a remarkable increase of heat transfer performance over the base fluid for the same Reynolds number.

Numerical investigations on nanofluids were carried out by two approaches. The first approach assumes that the continuum assumption is still valid for fluids with suspended nano-size particles. The other approach uses a two-phase model for better description of both the fluid and the solid phases.

The single phase model, with thermophysical properties all assumed to be constant with temperature, was employed in [21-24].

The two phase approach seems to be a better model to describe the nanofluid flow. In fact, the slip velocity between the fluid and particles may not be zero [10] due to several factors such as gravity, friction between the fluid and solid particles, Brownian forces and thermophoresis. The two phase approach provides a field description of the dynamics of each phase or, alternatively, the Lagrangian trajectories of individual particles coupled with the Eulerian description of the fluid flow field [25-30].

As can be seen, all the aforementioned literature is focused on the theoretical, experimental and numerical study of thermophysical properties and convection of nanofluids, but the modern design concept for a thermal system, pursues not only the enhancement of heat transfer performance, but also requests the minimal power requirements.

Enhancement of the heat transfer performance, usually, must be achieved at the expense of power input and this is also the case of nanofluids. In fact, in the study of nanofluid convection, there is the recurrent question of where is the position of the trade-off between the increase in heat transfer and pressure loss. Therefore, the optimal trade-off between heat transfer and power input requirement becomes a major issue in the design of a thermal system.

A modern approach for the optimization of a thermal system is based on the second law of thermodynamics. Particularly, the entropy generation is used as the parameter for evaluating the efficiency of the system. The system with minimum entropy generation is considered as the optimal design [31,32].

In our opinion, an accurate way to handle this common problem is to analyze the entropy generation in order to ascertain the condition under which entropy generation is minimized.

In this paper, developing turbulent forced convection flow of a nanofluid in a channel with square transversal section is numerically investigated. Steady state of a two-dimensional symmetric flow is considered and the channel is heated at uniform heat flux. The study is carried out for water with alumina particles with a spherical size of 38 nm diameter. The main aim of the present work is to estimate the thermal and fluid flow fields and to find, by means of second law analysis, the channel optimal working condition under given boundary conditions and particles' concentration. An analytical procedure is also proposed to estimate the entropy generation and a comparison with the numerical results is accomplished.

To the authors' best knowledge, it seems that nanofluids forced convection in tubes, with square section in turbulent regime, has not been previously investigated. Moreover, it seems that the optimization by means of second law analysis is applied for the first time to nanofluids convection. The intention of this investigation is to try to bridge the information gap.

## Mathematical modelling

**Figure 1:**
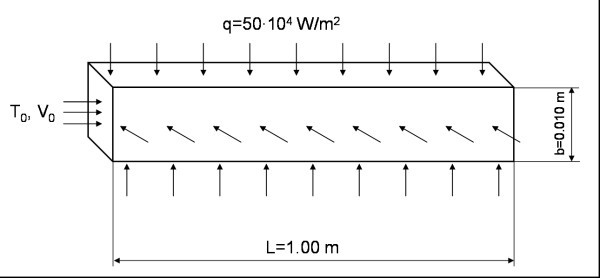
**Geometrical configuration under investigation**.

A sketch of the considered geometrical configurations is reported in Figure 1. The tube with square section has a length *L *equal to 1.00 m, the side *b *is equal to 0.010 m. These values allow to obtain a fully developed flow both dynamically and thermally at the outlet section. The nanofluid under consideration is composed of water and particles of Al_2_O_3 _with spherical size and a diameter equal to 38 nm.

Boundary conditions and geometry are symmetrical; flow and thermal fields are assumed to be symmetrical with respect to the vertical plane passing through the tube main axis; half computational domain is, therefore, considered.

### Governing equations

In the present article, the thermal and fluid dynamic behavior of an Al_2_O_3_/water nanofluid is analyzed by means of the mixture model, which seems to give accurate results with nanofluids [26,27,29,30]. In the mixture model, particles are taken into account by adding a term in the momentum conservation equation and solving the concentration equation.

Each phase has its own velocity vector, and within any control volume, there is a fraction of each phase, in accordance with the space occupied by the base fluid and particles.

The governing equations in mathematical formulation of the mixture model for average steady-state conditions in dimensional form, following the analysis given in [25-27,29,30,33], are:

Conservation of mass:(1)

Momentum equation:(2)

Volume fraction:(3)

Conservation of energy:(4)

The compression work and the viscous dissipation are assumed negligible in the energy equation, Equation 4.

In the conservation of momentum, Equation 2,  is the drift velocity for the secondary phase *k*, i.e. the nanoparticles in the present study:(5)(6)(7)

The slip velocity is the relative velocity of the secondary phase, nanoparticles, with respect to the primary phase, fluid. It is:(8)

The drift velocity and relative velocity are linked by:(9)

The relative velocity is evaluated by the equation proposed in [33]:(10)

whereas the drag function is calculated by means of the following equation:(11)

proposed by Schiller and Naumann [34].

The acceleration in Equation 10 is given by(12)

### Turbulence modeling

The *k*-ε model, proposed by Launder and Spalding [35], is employed to close turbulence model. The model introduces one equation for the turbulent kinetic energy, *k*, and another equation for the rate of dissipation, ε. To take into account the presence of nanoparticles, the following formulation is considered as suggested in [26,29]:(13)(14)

where(15)(16)

with *C*_1 _= 1.44, *C*_2 _= 1.92, *C*_*μ *_= 0.09, *σ*_*k *_= 1, *σ*_*ε *_= 1.3.

### Nanofluids physical properties

The most difficult problem in nanofluids simulation is posed by the evaluation of thermophysical properties, particularly viscosity and thermal conductivity, because it is not clear if classical equations give reliable results. However, on the other hand, a few experimental data are available to build new models [26].

In the present article, the following equations are considered to evaluate Al_2_O_3_/water nanofluid thermophysical properties:(17)(18)(19)(20)

Equations 17 and 18 are based on the classical theory of two-phase mixture and given in [19,21-23,28,29]. Equation 18 was first employed in [19] and then utilized in many different articles [21-23,28-30]. Another formulation of specific heat, based on heat capacity concept, is present in literature, as reported in [21,36]. The maximum difference between the two formulations is about 10% for a particle concentration of 6%, whereas at lower concentration, φ = 1%, the deviation is about 2%. In order to understand if this difference is acceptable, the bulk temperature of the fluid is estimated by applying the first law of thermodynamics. As known from basic thermodynamics, *C*_p _represents the amount of heat necessary to increase the temperature of a substance of one degree. This implies that the main effect of *C*_p _should be noticed on the bulk temperature of the fluid. In accordance with this, using the two different formulations of *C*_p_, the variation on the bulk temperature is estimated to be about 3% at *Re *= 20 × 10^3 ^and it reduces at the increase of *Re*. Therefore, from an engineering point of view, the two approaches can be considered to yield the same results.

Equation 19 was proposed in [21-23,30] and obtained as a result of a least square curve fitting of available experimental data [9,37,38] for the considered mixture. To assess their consistency, the results obtained from Equation 19 are compared with the very recent model proposed in [39]. The comparison show a difference of -0.4, -0.4 and -6.6% for concentrations of 1, 4 and 6%, respectively. Therefore, it can be concluded that Equation 19 gives, for the present case, a valid estimation of nanofluid viscosity. As for thermal conductivity, Equation 20 was obtained in [21-23] using the well known model proposed by Hamilton and Crosser [17], assuming spherical particles. Such a model, which was first developed on data from several mixtures containing relatively large particles, i.e. millimetre and micrometre size particles, is believed to be acceptable for use with nanofluids. In order to prove this, the results of Equation 20 are compared with the model proposed in [14], showing a maximum deviation of -2.5% for a concentration of 6%. In light of the small difference, Equation 20 is to be considered a valid formulation to estimate nanofluid thermal conductivity in the present case.

It should be noted that the validity of the reported Equations 17 to 20 remains a subject of debate. At present, there is no agreement in the nanofluids community about the description of thermophysical properties [26].

In the present article, the thermophysical properties considered for Al_2_O_3 _are [37]:

and those of base fluid are:

### Entropy generation analysis

The total entropy generation in the considered channel is obtained as [31]:(21)

In Equation 21, two contributions represent the entropy generation due to heat transfer and the friction losses, respectively. To understand the weight of each contribution to the entropy generation a dimensionless parameter, the Bejan number (*Be*), is considered:(22)

The value of *Be *ranges from 0 to 1. Accordingly, *Be *= 0 and *Be *= 1 are two limiting cases representing the irreversibility is dominated by fluid friction and heat transfer, respectively. The two quantities on the second member of Equation 21 are expressed, according to Bejan [31], as follows:(23)

Analyzing Equation 23, it becomes evident that a high Stanton number contributes to the reduction of the heat transfer share of *S*_gen_, (*S*_gen_)_T_, whereas a high friction factor has the effect of increasing the entropy generation rate due to viscous effects, (*S*_gen_)_F_. Despite its simple form, Equation 23 is a very powerful tool because it allows to minimize the entropy generation inside a given channel with a determined nanofluid subjected to a known heat flux, which is quite a common case.

If nanofluid, channel and heat flux are given, all the thermophysical properties, geometrical factors and heat power are known. The only unknown variables in Equation 23 are Stanton number, mass flow rate, *T*_b,av _and the friction factor. Except for *T*_b,av_, which needs to be estimated as shown in the following, the others parameters, if an adequate correlations for *Nu *is introduced in *St *and a proper correlation is used for *f*, are all function of the velocity.

In the present investigation, the correlation proposed by Pak and Cho [19] is used to calculate *Nu*(24)

and, according to [19], the correlation proposed by Kays and Crawford [40] is used to evaluate *f*(25)

Combining Equations 24, 25 with Equation 23 and setting ∂*S*_gen_/∂*V *= 0, it is possible to calculate the optimal velocity to minimize entropy generation:(26)

Once Equation 26 is known, the *Re*_opt _value is evaluated as:(27)

The bulk temperature is estimated by means of an energy balance on the inlet and outlet section of the tube:(28)

From Equation 28, it is possible to calculate the outlet temperature and re-arrange it in terms of *Re*, to obtain the following expression:(29)

With *T*_out _known the bulk temperature of the fluid can be determined as following [41]:(30)

Equations 27 and 30 are coupled, hence they are solved by successive iterations, until the chosen convergence criteria is respected. In the present analysis, the solution is considered to converge if the variation of the bulk temperature between steps *n *and *n *- 1 is less than 0.001%.

The results of the present analytical formulation are reported in Table 1.

**Table 1:** Optimal values of Reynolds number, determined from Equation 27, in order to minimize entropy generation.

	φ = 1%	φ = 4%	φ = 6%
*Re*_opt_	89 × 10^3^	68 × 10^3^	56 × 10^3^

### Boundary conditions

In the following analysis, uniform axial velocity and temperature profiles are assigned and constant turbulence intensity, *I*, is imposed at the channel inlet. The inlet temperature and turbulence intensity value are *T*_0 _= 293 K and *I*_0 _= 1%, respectively, in all considered cases.

At the channel exit section, the fully developed conditions are obtained, that is to say that all axial derivatives are zero. On the channel wall, the non-slip conditions and a uniform heat flux (*q *= 50·10^4 ^W m^-2^) are imposed. Moreover, both turbulent kinetic energy and dissipation are equal to zero. Flow and thermal fields are assumed symmetrical with respect to the vertical plane passing through the channel longitudinal axis. Therefore, a symmetry boundary condition is applied on the aforementioned plane, that is to say the gradient of all variables is zero.

### Numerical method and validation

The computational fluid dynamic code Fluent [42] was employed to solve the present problem. The governing equations (Equations 1 to 4) were solved by control volume approach. The residuals resulting from the integration of the governing equations (Equations 1 to 4) are considered as convergence indicators. Convergence is considered achieved, when the residuals of Equations 1 to 4 are in the order of 10^-6^, 10^-8^, 10^-8 ^and 10^-8^, respectively.

In order to ensure the accuracy as well as the consistency of numerical results, three non-uniform grids were subjected to an extensive testing procedure.

**Figure 2:**
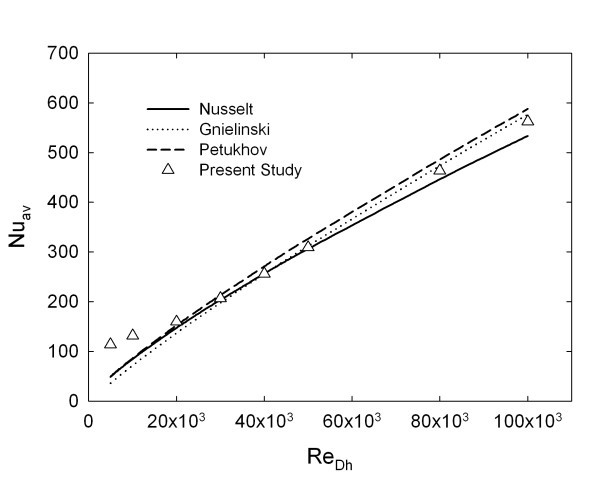
**Comparison of average Nusselt number of this study with correlations proposed by Gnielinski **[41]**, Petukhov **[42]**and Nusselt **[43].

Results showed that for the problem under consideration, the chosen non-uniform grid seems to be sufficient to guarantee the precision of numerical results and their independency with respect to the number of elements used. The considered grid has 25, 50 and 200 elements along the horizontal, vertical and axial directions, respectively, with heavily packed grid points close to the channel wall and at the entrance region, where the temperature and velocity gradients are significant [29]. The computational grid is validated using the correlations proposed by Gnielinski, as also suggested in [27], Petukhov and Nusselt [43-45] for pure water, as shown in Figure 2.

As stated in previous sections, it is assumed that the heat exchange happens by means of forced convection and the contribution of natural convection is not taken into account. In order to show the validity of this assumption, the quantity *Gr *⋅ *Pr *is evaluated for *Re *= 5 × 10^3^, 10 × 10^3 ^and 20 × 10^3 ^at *z*/*D *= 50, obtaining the following results: (approx.) 5.0 × 10^6^, 4.0 × 10^6 ^and 3.0 × 10^6^, respectively. These results are compared with the data reported in [46], showing that for *Re *= 10 × 10^3 ^and 20·10^3 ^there is no contribution of natural convection at all, whereas *Re *= 5 × 10^3 ^seems to be a limit condition, and the contribution of natural convection might be neglected without loosing too much accuracy. It is not possible from the data presented in [46] to understand exactly what happens at *Re *= 5 × 10^3^, because the corresponding value of *Gr *⋅ *Pr *is out of the considered range. For the purpose of this analysis, the contribution of natural convection can be neglected without loosing too much accuracy.

## Results

Results were carried out employing the mixture model, for φ = 1, 4 and 6, *Re*_Dh _= 5 × 10^3^, 1 × 10^4^, 2 × 10^4^, 3 × 10^4^, 4 × 10^4^, 5 × 10^4^, 8 × 10^4 ^and 1 × 10^5^, and *q *= 5 × 10^5 ^W m^-2^. In all cases, the particles size is considered equal to 38 nm.

### Thermal and fluid fields discussion

First, it is of fundamental importance to understand the impact of nanoparticles on the turbulent flow. As remarked in [10], an important concept in turbulent flow is that of 'energy cascade'. That is, the kinetic energy originated by the turbulence goes first into larger eddies, from which it is transferred to smaller eddies, then into further smaller ones, until it is converted to heat by viscous forces. Given these facts, it is fundamental to understand if the nanoparticles can conflict with this energy exchange, thereby suppressing turbulence. As observed in [10], considering a turbulent flow inside a tube of equivalent diameter *D *and mean velocity , the length scale of the large eddies, *l*_0_, would be of the order of *D*, and their time scale, *t*_0_, of the order of *D*/. By means of the Kolmogorov's scaling laws [47], it is possible to determine the length scale, *l*_s_, and time scale, *t*_s_, of the smallest eddies:(31)(32)

In the case of *Re *equal to 1 × 10^5^, *l*_s _~ 2 μm and *t*_s _~ 2 μs (for particle concentration equal to 6%) are obtained. Therefore, the length and time scales of turbulent eddies are much larger than the nanoparticle size, 38 nm, and relaxation time, ~2 ns, estimated as suggested in [10]. This means that the nanoparticles are transported very effectively by the turbulent flow.

**Figure 3:**
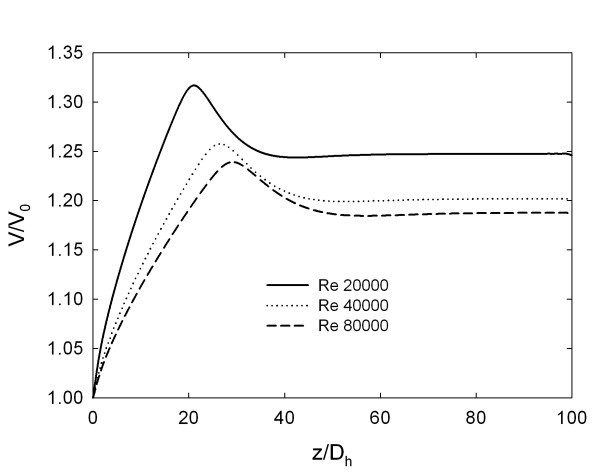
**Axial evolution of centreline velocity for φ = 4%**.

The development of the axial velocity along the tube centreline for φ = 4% is shown in Figure 3 and the results suggest the existence of a fully developed region for *z*/*D *≈ 40 for all the considered Reynolds numbers. Immediately after the tube inlet, the boundary layer growth pushes the fluid towards the centreline region, causing an increase of the centreline velocity. As the Reynolds number increases, the maximum value of axial velocity moves further downstream, because the increase of axial momentum transports the generated turbulence in the flow direction. After the maximum point, the velocity at the centreline decreases in order to respect the continuity equation, as also reported in [29].

It is interesting to note that the maximum and fully developed values of the dimensionless centreline velocity decrease as Reynolds number increases. This effect is due to the fact that the corresponding velocity profiles become more uniform as *Re *increases.

**Figure 4:**
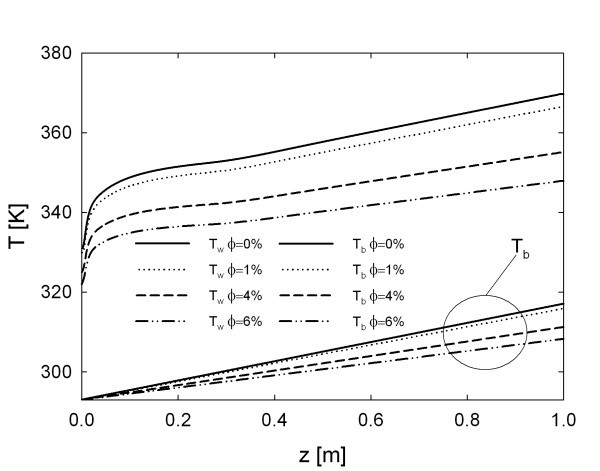
**Effect of particle loading, for *Re *= 20 · 10**^**3**^**, on the axial development of wall and bulk temperature**.

In Figure 4, wall and bulk temperature profiles for *Re *= 20 × 10^3 ^are reported. The figure clearly shows that the inclusion of nanoparticles has a beneficial effect on the wall temperature, which decreases according to increase in the particles' concentration.

Particularly, the temperature difference at tube exit, between base fluid and nanofluid with φ = 6% is about 20 K. Consequently, particles effect is remarkable. This behaviour can be explained by means of the higher thermal capacity (i.e. the product of density and heat capacity) of nanofluids with respect to conventional fluid. Therefore, more energy is required to increase the bulk temperature. This property is of fundamental importance, because it allows to downsize devices without violating thermal constraints, as shown in [47,48].

**Figure 5:**
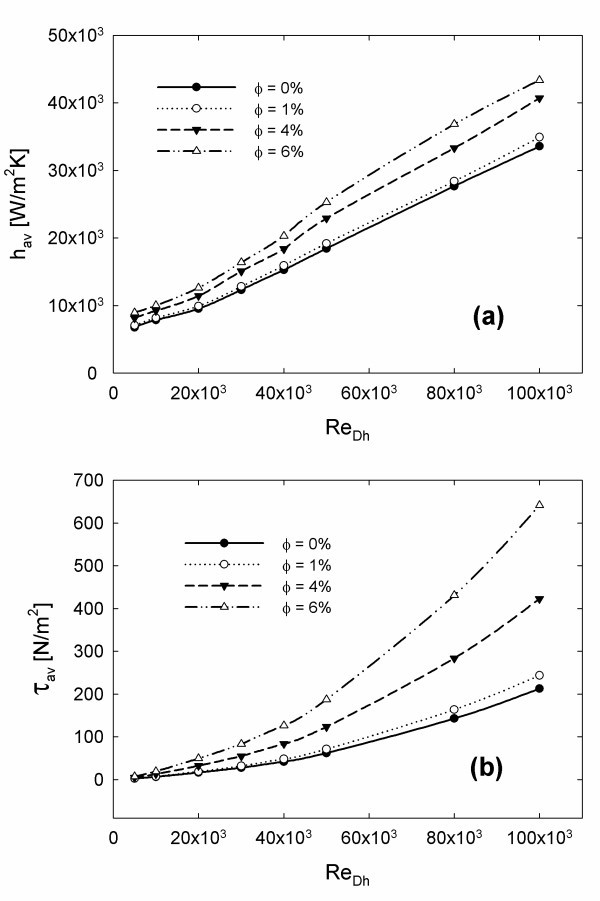
**Effect of Reynolds number and particles' concentration on: (a) average heat transfer coefficient; (b) average wall shear stress**.

Increasing particles' concentration causes the increase of the average heat transfer coefficient, as clearly shown by Figure 5a.

The average shear stress on the tube wall is reported in Figure 5b. The figure shows an increase in the shear stress in accordance with the concentration and Reynolds number. For the lowest concentration considered, φ = 1%, the increase in the shear stress with respect to the base fluid is about the 10%, while for the other concentration values, φ = 4 and 6%, the increment is noticeable and it increases in accordance with concentration and Reynolds number. As for the friction factor, it is observed to be in strong agreement with Equation 25, but for the sake of brevity, the relative diagram is not reported. It is important to underline that looking at Figure 5a, b it is not possible to make deductions about the energetic convenience in using of nanofluids.

**Figure 6:**
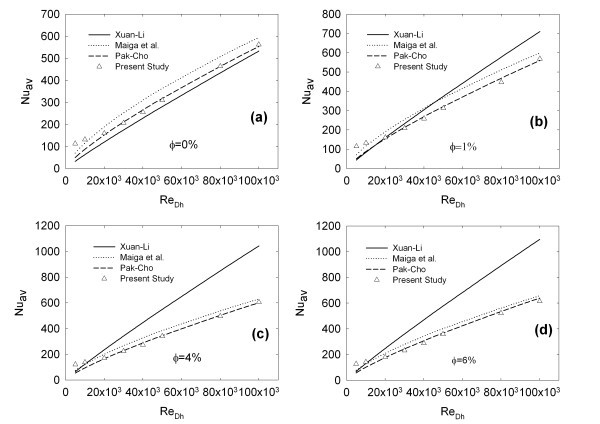
**Average Nusselt number comparison with the correlations proposed by Pak and Cho **[19]**, Xuan and Li **[20]**and Maiga et al. **[22]**for (a) φ = 0%, (b) φ = 1%, (c) φ = 4% and (d) φ = 6%**.

Average Nusselt number, for all the concentrations and Reynolds numbers, is reported in Figure 6. In this figure, comparisons with experimental and numerical correlations, present in the literature, are also provided.

A comparison with the experimental correlations proposed by Pak and Cho [19] and Xuan and Li [20] and the numerical correlation proposed by Maiga et al. [22] is accomplished.

Figure 6a shows the performances of the aforementioned correlations when only the base fluid is considered. The correlation proposed by Maiga et al. [22] over-estimates the values provided by Pak and Cho [19] by about 20%, while Xuan and Li [20] correlation under-estimates them by about 15%. However, these results can be considered acceptable, as reported also by Buongiorno [10].

Average Nusselt number for φ = 1% is reported in Figure 6b. The values of the present work are in very good agreement with Pak and Cho [19] correlation, except that for *Re *= 5.0 × 10^3 ^and 10 × 10^3^, which fits the value given by Maiga et al. [22] However, it is important to remark that *Re *= 10 × 10^3 ^is the lowest limit to apply Pak and Cho correlation, which was obtained in the range 10 × 10^3 ^<*Re *< 100 × 10^3 ^[19].

For *Re *< 30 × 10^3^, Xuan and Li [20] data are in agreement with Pak and Cho [19] and the present numerical data, whereas for *Re *> 30 × 10^3^, there is a deviation, which leads to over-estimated values. For φ = 4% and φ = 6%, in Figure 6c, d, respectively, the average Nusselt number presents a similar behaviour to the previous case. In fact, the average Nusselt numbers of the present work are in strong agreement with the results of Pak and Cho [19] except that for *Re *= 10 × 10^3 ^which, on the contrary, is in agreement with Maiga et al. [22] and Xuan and Li [20] and *Re *= 5 × 10^3^, which is greater than all the correlations values. For *Re *> 2.0 × 10^4^, Xuan and Li's[20] correlation significantly overestimates the average Nusselt number.

It is important to note, as also indicated by Buongiorno [10], that Pak and Cho's [19] correlation is completely empirical, whereas Xuan and Li's [20] is based on the dispersion model, but it needs five empirical coefficients to match the data. They experimentally determined these five coefficients to match their analytical correlation. This fact could explain the deviation between the two earlier mentioned correlations. Moreover, it is important to remark that Pak and Cho [19] developed their correlation working with Al_2_O_3 _nanofluid, as in the present article, whereas Xuan and Li [20] worked with Cu nanofluid, even though their correlation should be valid in general [20,33].

### Entropy generation discussion

In this section, the entropy generation analysis is presented. The analysis is formulated in global terms and it allows to understand the optimal working conditions for the considered channel from the energetic point of view.

**Figure 7:**
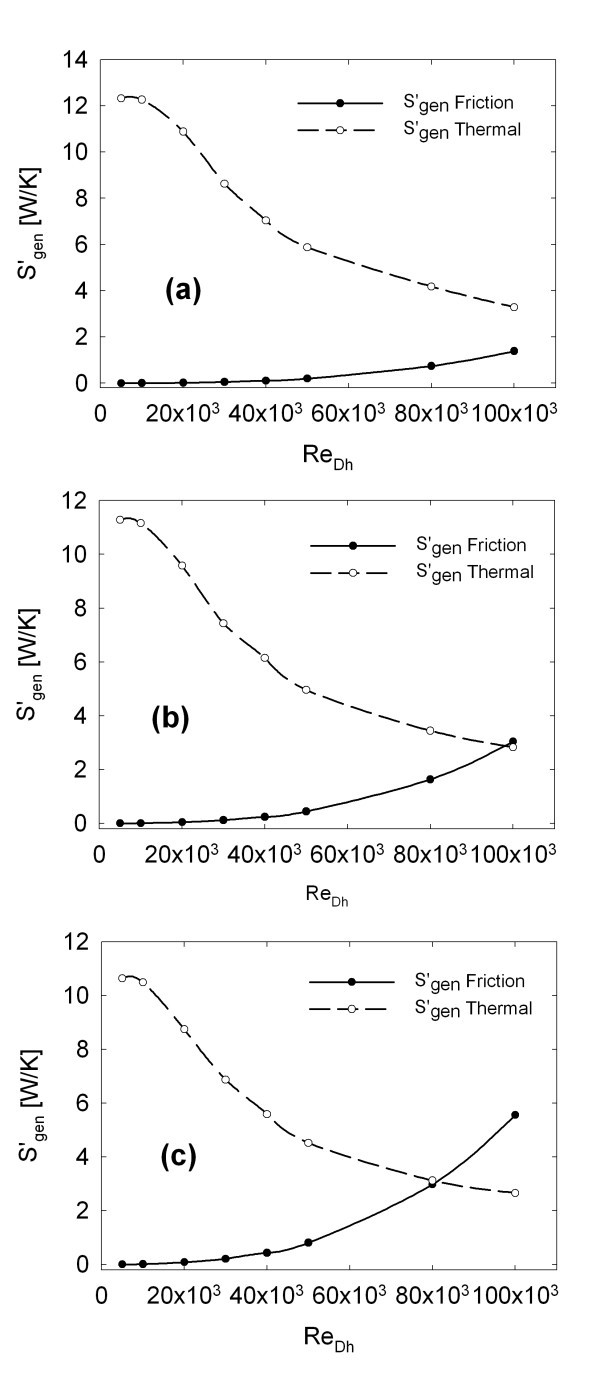
**Entropy generation due to heat transfer irreversibility and friction losses for (a) φ = 1%, (b) φ = 4%, (c) φ = 6%**.

Entropy generation due to heat transfer and friction losses is reported for each concentration, as a function of Reynolds number in Figure 7, for φ = 1%, Figure 7a, φ = 4%, Figure 7b, and φ = 6%, Figure 7c. It is possible to observe that as *Re *value increases, there is a reduction of (*S*_gen_)_T_, because there is a decrease in the difference between wall and bulk average temperatures, which causes a decrease in the entropy generation. On the contrary, as *Re *increases, there is an increment of (*S*_gen_)_F_, because of the higher values of velocity gradient, which causes an increase of the wall shear stress, and, consequently, of the friction losses.

As the particles' concentration increases, (*S*_gen_)_T _decreases and (*S*_gen_)_F _increases. This happens because a higher particles' concentration improves the heat transfer between wall and fluid contributing to a reduction in the difference between wall and bulk temperature. However, it also causes an increase of the nanofluid viscosity, which leads to an increase of the shear stress.

**Figure 8:**
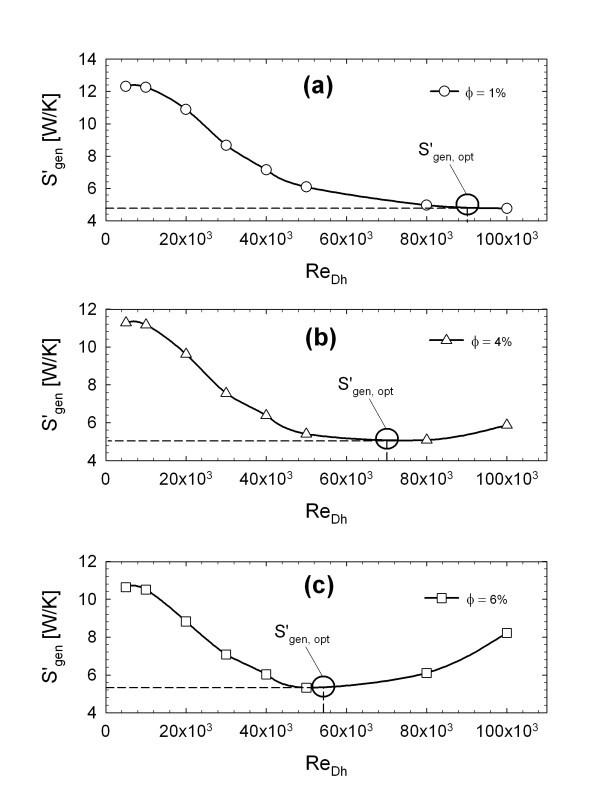
**Total entropy generation for (a) φ = 1%, (b) φ = 4%, (c) φ = 6%**.

According to Equation 27, the optimal Reynolds number for each concentration is calculated, as reported in Table 1. It is noted that *Re*_opt _value decreases as the φ value increases. This is also shown in Figure 8, where the numerical calculation of total entropy generation as a function of *Re *is reported for each concentration value. The analytical results of Equation 27 are in very good agreement with the numerical results (Figure 8); in fact, the minimum points of the curves correspond to the Reynolds values reported in Table 1. This result can also be considered an indirect proof of the agreement between results proposed by Pak and Cho [19] and the mixture model employed in the present article. In Figure 8, it is noted that the optimal value of Reynolds number decreases as the concentration increases. This happens because the increase of the viscosity becomes more and more important, overcoming the beneficial effect that the particles have on the heat transfer and, consequently, on (*S*_gen_)_T_.

**Figure 9:**
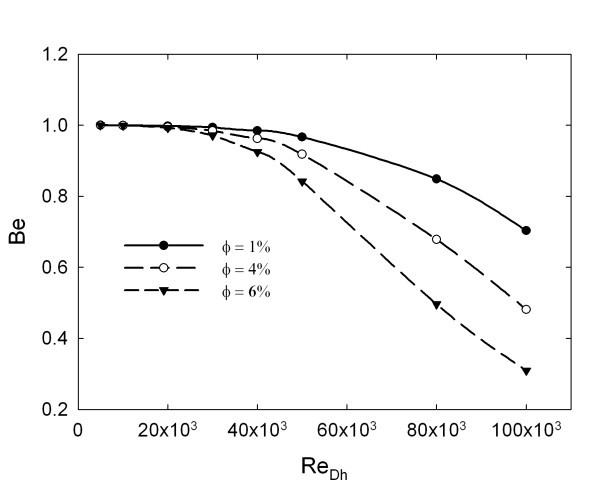
**Bejan number for different φ values: 1, 4 and 6%**.

The behaviour of Bejan number is reported in Figure 9. It is observed that Be decreases as Re and φ increase, showing that (*S*_gen_)_F _is increasing. For φ = 4% and *Re *= 1.0 × 10^5 ^and for φ = 6% and Re = 8.0 × 10^4^, *Be *is about 0.5. This means that entropy generation, due to heat transfer and friction losses, have the same weight. Up to *Re *= 2 × 10^4^, *Be *is equal to 1 for all concentrations, showing that in all considered cases, the entropy generation is due to thermal irreversibility. For *Re *> 2 × 10^4^, *Be *value starts to decrease, but with different slopes according to particles' concentration. Particularly, at the higher concentration, there is higher slope, because the friction losses, due to the increase of *Re *and viscosity, become more relevant.

## Conclusions

In this article, the hydrodynamic and thermal behaviours of water-Al_2_O_3 _nanofluids, flowing inside uniformly heated tubes with square section, were numerically investigated, at average steady state-conditions, in turbulent flow. The entropy generation analysis was performed in order to determine the optimal working condition for the given geometry under the considered boundary conditions. The mixture model with constant temperature properties was employed to simulate the nanofluid.

Results clearly showed that, the inclusion of nanoparticles produced a considerable increase of the heat transfer with respect to that of the base liquid. Heat transfer enhancement increased with the particle volume concentration, but it was accompanied by increasing wall shear stress values. For each investigated concentration value, the enhancement was higher for the highest Reynolds number considered and a very good agreement was found with the experimental data from Pak and Cho [19]. Accordingly Pak and Cho's correlation can also be used for square section tube, if the hydraulic diameter is used.

The optimal Reynolds number was analytically determined, in terms of minimum entropy generation value, and it is in very good agreement with the numerical results. The entropy generation analysis has shown that, at low *Re *value, the entropy generation, due to the irreversibility of heat transfer, dominates, whereas with increasing Re value and particles' concentration, the entropy generation, due to friction losses, becomes more important. The optimal value of *Re *decreases as particles' concentration increases.

## List of symbols

*a*: acceleration (m s^-2^); *A*: cross section area (m^2^); *b*: square side (m); *Be*: Bejan number (Equation 22); *C*p: specific heat (J kg^-1 ^K^-1^); *d*: particles diameter (m); *D*_h _: hydraulic diameter, (m); *f*: friction factor; *f*_drag_: drag function; *F*: force (N); *g*: gravitational acceleration (m s^-2^); *G*: mass velocity, (*G *= *ṁ*/*A*) (kg s^-1 ^m^-2^); *Gr*: Grashof number, ; *h*: heat transfer coefficient (W m^-2 ^K^-1^); *H*: enthalpy (J); *k*: turbulent kinetic energy (m^2 ^s^-2^); *I*: turbulence intensity; *L*: channel length (m); *ṁ*: mass flow rate (kg s^-1^); *Nu*: Nusselt number, *Nu *= *hD*_h_/*k*_0_; *p*: pressure (Pa); *Pr*: Prandtl number, *Pr *= *C*_p_μ/*k*; *q*: wall heat flux (W m^-2^); *q*": heat transfer per unit length (W m^-1^); *Q*: thermal power (W); *Re*: Reynolds number, *Re *= *V*_0_*D*_h_/μ; *S*_gen_: entropy generation per unit length (W K^-1 ^m^-1^); *St*: Stanton number, *St *= *h*_av_/(*C*_p _⋅ *G*); *T*, *t*: time-mean and fluctuating temperature (K); *V*, *v*: time-mean and fluctuating velocity (m s^-1^); *y*: transversal coordinate (m); *z*: axial coordinate (m); *Greek letters *β: coefficient of thermal expansion (K^-1^); ε: dissipation of turbulent kinetic energy (m^2 ^s^-3^); φ: particle volume concentration; λ: thermal conductivity of the fluid (W m^-1 ^K^-1^); ν: kinematics viscosity (m^2 ^s^-1^); μ: dynamic viscosity (kg m^-1 ^s^-1^); ρ: density (kg m^-3^); τ: wall shear stress (Pa); *Subscripts *av: average value; b: bulk value; eff: effective; f: primary phase (base fluid); F: friction; *k*: *k*th phase; m: mixture (nanofluid); opt: optimum; out: outlet section; p: particle property; r: nanofluid/base-fluid' ratio; t: turbulent; T: heat transfer; w: channel wall; 0: inlet condition

## Competing interests

The authors declare that they have no competing interests.

## Authors' contributions

VB carried out all the numerical and analytical analysis and prepared the first draft of the manuscript. VB, OM and SN revised the manuscript improving the comments and the form.
